# Complex effects of flavopiridol on the expression of primary response genes

**DOI:** 10.1186/1747-1028-7-11

**Published:** 2012-03-29

**Authors:** Havva Keskin, Judit Garriga, Daphne Georlette, Xavier Graña

**Affiliations:** 1 Fels Institute for Cancer Research and Molecular Biology, Temple University School of Medicine, AHP bldg., room 308, 3307 North Broad St, Philadelphia, PA 19140, USA; 2 Department of Biology, Temple University, 436 Biology Life Sciences Building, Philadelphia, PA 19122, USA; 3 Department of Biochemistry, Temple University School of Medicine, Philadelphia, PA 19140, USA

**Keywords:** Primary Response genes, Mitogenic stimuli, Quiescence, Transcription, CDK9, RNA polymerase II, CDKs, Control of gene expression

## Abstract

**Background:**

The Positive Transcription Elongation Factor b (P-TEFb) is a complex of Cyclin Dependent Kinase 9 (CDK9) with either cyclins T1, T2 or K. The complex phosphorylates the C-Terminal Domain of RNA polymerase II (RNAPII) and negative elongation factors, stimulating productive elongation by RNAPII, which is paused after initiation. P-TEFb is recruited downstream of the promoters of many genes, including primary response genes, upon certain stimuli. Flavopiridol (FVP) is a potent pharmacological inhibitor of CDK9 and has been used extensively in cells as a means to inhibit CDK9 activity. Inhibition of P-TEFb complexes has potential therapeutic applications.

**Results:**

It has been shown that Lipopolysaccharide (LPS) stimulates the recruitment of P-TEFb to Primary Response Genes (PRGs) and proposed that P-TEFb activity is required for their expression, as the CDK9 inhibitor DRB prevents localization of RNAPII in the body of these genes. We have previously determined the effects of FVP in global gene expression in a variety of cells and surprisingly observed that FVP results in potent upregulation of a number of PRGs in treatments lasting 4-24 h. Because inhibition of CDK9 activity is being evaluated in pre-clinical and clinical studies for the treatment of several pathologies, it is important to fully understand the short and long term effects of its inhibition. To this end, we determined the immediate and long-term effect of FVP in the expression of several PRGs. In exponentially growing normal human fibroblasts, the expression of several PRGs including FOS, JUNB, EGR1 and GADD45B, was rapidly and potently downregulated before they were upregulated following FVP treatment. In serum starved cells re-stimulated with serum, FVP also inhibited the expression of these genes, but subsequently, JUNB, GADD45B and EGR1 were upregulated in the presence of FVP. Chromatin Immunoprecipitation of RNAPII revealed that EGR1 and GADD45B are transcribed at the FVP-treatment time points where their corresponding mRNAs accumulate. These results suggest a possible stress response triggered by CDK9 inhibition than ensues transcription of certain PRGs.

**Conclusions:**

We have shown that certain PRGs are transcribed in the presence of FVP in a manner that might be independent of CDK9, suggesting a possible alternative mechanism for their transcription when P-TEFb kinase activity is pharmacologically inhibited. These results also show that the sensitivity to FVP is quite variable, even among PRGs.

## Background

The Positive Transcription Elongation Factor b (P-TEFb) is a complex of CDK9 and either cyclins T1, T2 or K [1-4]. P-TEFb is recruited to promoters by transcription factors and/or BRD4 where it stimulates transcriptional elongation by phosphorylating the C-terminal domain (CTD) of RNA polymerase II (RNAPII) and the negative elongation factors DSIF and NELF [5-7]. Although cyclin K was first identified as a CDK9 partner, it appears to prefer CDK12 and CDK13, which also play a role in elongation [8]. Yet, a role for cyclin K/CDK9 has been recently reported in maintaining genomic integrity [9,10]. Recent work has lead to the proposal that CDK9 and CDK12/13 are the orthologs of yeast Bur1 and CTK1, respectively [8], which play distinct roles in elongation by RNAPII.

Flavopiridol (FVP) is a potent inhibitor of CDKs, with significant selectivity for CDK9, as its IC50 has been found to be about 7 times lower than that of the closest CDK IC50 reported to date [11]. Incubation of HeLa or 293 cells for one hour with 300 nM FVP inhibits transcription by 60-70%, as measured in run-on assays [12,13]. At a concentration of 1 μM, treatment of OCI-Ly3 cells with FVP results in rapid downregulation of genes with mRNA microarray patterns similar to those obtained with the transcriptional inhibitor Actinomycin D, and the kinetics of mRNA downregulation reflect the half life of the mRNAs measured [14]. However, when human T98G cells and BJ-TERT fibroblasts were treated with 300 nM FVP for extended periods of time (4 to 24 h) or with a dominant form of CDK9, we observed upregulation of a very significant number of mRNAs [15]. Among the genes that were upregulated were a number of primary response genes (PRGs). PRGs are genes that are induced in response to a variety of signals and do not require *de novo *protein synthesis [16]. Our findings appeared to be at odds with recent work that has shown that expression of PRGs responsive to LPS stimulation in macrophages correlates with recruitment of cyclin T1 and CDK9 to those genes coinciding with phosphorylation of the CTD of RNAPII on Ser-2, and that preincubation of macrophages with 5,6-dichloro-1-beta-D-ribofuranosyl-benzimidazole (DRB), a CDK9 inhibitor, prevents RNAPII Ser-2 phosphorylation and productive elongation of these genes [17].

Since inhibition of CDK9 activity is being explored as a therapeutic avenue in a number of diseases including AIDS, cancer, cardiac myopathies and inflammatory processes [11,18-20], it is important to examine the time dependent consequences of CDK9 inhibition on gene expression. To this end, we have determined the short and long term effects of FVP treatment on the expression of PRG mRNAs and the localization of RNAPII in these genes. Our results suggest that a fraction of PRGs are transcribed in the presence of FVP, under conditions where phosphorylation of the CTD of RNAPII on Ser-2 and Ser-5 and the expression of other genes are clearly inhibited.

## Results

We have found that a large number of genes are upregulated after treating T98G cells [15] or hTERT-immortalized normal human fibroblasts (BJ-TERT fibroblasts) (unpublished observations) with flavopiridol (FVP) for 4 h or more. Surprisingly, some of these genes are classical PRGs. Because the normalized data set for the BJ-TERT fibroblasts study is much larger, all experiments and analyses described below were performed using BJ-TERT fibroblasts (the results of the entire BJ-TERT dataset will be reported elsewhere).

**Figure 1:**
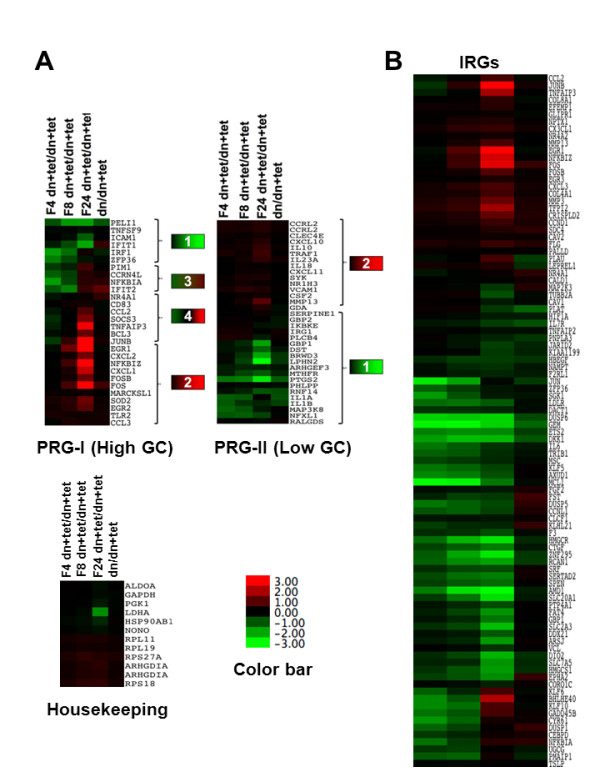
**Effect of FVP on the expression of PRGs and IRGs in BJ-TERT fibroblasts. (A) **BJ-TERT fibroblasts were infected with tet-off and dnCDK9 adenviruses (dn) in the presence or absence of tetracyclin (tet) and treated with FVP (F) as indicated. FVP treated cells were infected with control adenoviruses (dn + tet) to eliminate viral transduction as a variable in the comparison to dnCDK9 expressing cells. The expression ratios of FVP (F) or dnCDK9 (dn) vs. control adenoviruses in the presence of tet (dn + tet) were obtained. Hierarchical clustering was performed for PRG-I (top left), PRG-II (top right) and housekeeping genes (bottom) and visualized with Java TreeView. PRG-I group consists of 4 clusters that represent downregulated (cluster 1), upregulated (cluster 2) and early downregulated/later upregulated (cluster 3 and 4) genes. PRG-II group consists of 2 clusters (clusters 1 and 2), which behave similar as clusters 1 and 2 of PRG-I group. **(B) **Hierarchical clustering of Immediate Response Genes (IRGs) to mitogenic stimuli in BJ-TERT fibroblasts treated with FVP or dnCDK9 (gene arrays are in the same order as in A). A color gradient legend indicated fold changes in expression.

In BJ-TERT fibroblasts there were 2855 and 70 genes upregulated more than two fold with FVP treatment or dnCDK9, respectively. In the FVP dataset, this represents about one fourth of all genes modulated above the 2-fold cutoff, while the other three fourths are downregulated. Among the upregulated genes, were genes that are known to be regulated at the elongation phase of transcription such as JUNB, FOS and some primary response genes (PRGs) to LPS stimuli (Figure 1A and 1B). A recent study strongly suggests that P-TEFb is required for the expression of PRGs upon LPS stimulation of primary macrophages [17]. However, these results could be *a priori *seen in contrast with our microarray data that showed upregulation of several of these genes upon FVP treatment and in some cases with dnCDK9. Since Hargreaves and collaborators classified PRGs into two groups based on their GC promoter content: PRG-I (GC rich) and PRG-II (GC poor), we clustered genes designated PRG-I and PRG-II in their study using the expression log2 ratios from our microarray data obtained with BJ-TERT fibroblasts and visualized the results with Java-TreeView (Figure 1A). Among the PRG-I group, were genes downregulated upon FVP treatment at all time points and in some cases with expression of dnCDK9 (Figure 1A left, cluster 1). However, we also observed genes that were upregulated between 4 and 8 h of FVP treatment (Figure 1A left, cluster 2). It was also shown that another group of genes in clusters 3 and 4 were downregulated early but then were strongly upregulated at later time points (Figure 1A left, cluster 3 and 4). The PRG-II group included 2 different clusters of genes that behaved similarly to what was seen in the PRG-I group. Genes in cluster 1 were downregulated upon FVP treatment while genes in cluster 2 were upregulated (Figure 1A, right). Of note, visual examination of the expression of genes in the dnCDK9 array suggested a stronger correlation with the FVP arrays in the PRG-II group. This was confirmed by hierarchical subclustering of the PRGI and PRGII gene arrays shown in Figure 1A (Additional file 1: Figure S1). As a control, we clustered housekeeping genes typically used for mRNA expression normalization when measuring expression of specific mRNAs. Their expressions were mostly unaffected by FVP treatment or dnCDK9 with the exception of LDHA, which was downregulated by 24 h of FVP treatment (Figure 1A, bottom).

Also, genes previously defined as Immediate Response Genes (IRGs) to mitogenic stimuli [21] were clustered as shown in Figure 1B. In this group, most IRGs were downregulated with FVP and dnCDK9, but a significant number of "classical" IRGs such as EGR1, FOS, and CCND1, were upregulated upon FVP treatment, and in some cases by dnCDK9. Importantly, the expression profile of genes in clusters 3 and 4 of Figure 1A suggested that genes could be subject to inhibition by FVP at early time points and subsequently be upregulated, since the earliest FVP treatment time point in the microarray analysis was 4 h. Therefore, we rationalized that the genes in cluster 2 could potentially be downregulated prior to the 4 h time point collected. To test this possibility, a new set of time course experiments where BJ-TERT cells were treated with FVP for periods of time as short as 30 min was performed. The expression levels of these genes were determined by Q-RT-PCR as described in the Methods section.

### Q-RT-PCR experiments validate the microarray results and unveil genes whose expression is potently and rapidly inhibited by FVP and subsequently upregulated

For this analysis we selected the following PRG-I genes; EGR1, JUNB, and FOS (Figure 1A), and the IRG Cyclin D1 (Figure 1B), which are upregulated at 8 and 24 h of FVP treatment. We also selected the IRG response gene GADD45B, which is downregulated 4 and 8 h and subsequently upregulated at 24 h (Figure 1B). In addition, we selected two genes from the microarray analyses that were downregulated at all time points: HEXIM1 and MCL1. Of note, HEXIM1 is a known CDK9 target gene [22] and MCL1 is an IRG [21]. Finally, we also selected ALDOA, a housekeeping gene whose expression was found to be unchanged in the microarray analysis (Figure 1A, bottom panel).

**Figure 2:**
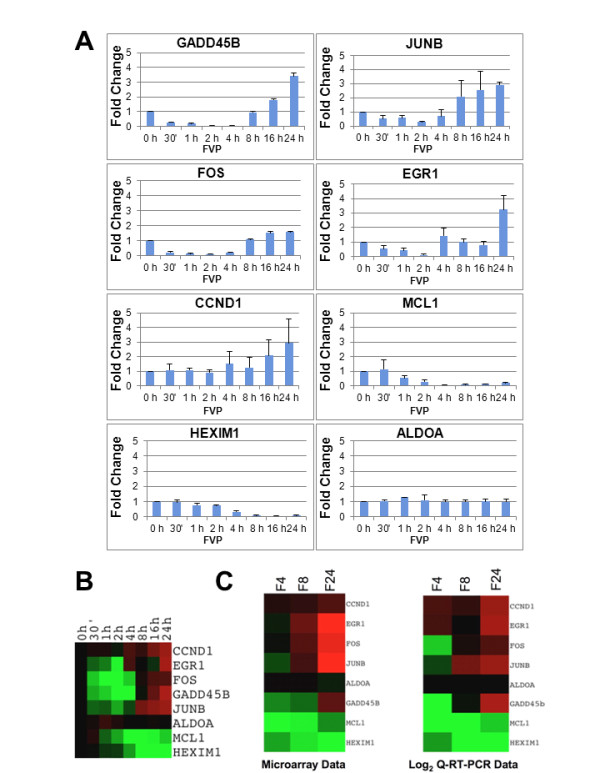
**Biphasic effect of FVP in the expression of certain PRG/IRGs. (A) **BJ-TERT fibroblasts were treated with 300 nM FVP as described in Figure 1A and the expression of indicated mRNAs was determined by Q-RT-PCR analysis. Data was represented as a fold change value with respect to mRNA expression at time zero. **(B) **Q-RT-PCR data was converted to log_2 _ratios and visualized with the same color scale used in microarray analysis using Java-TreeView. **(C) **Comparison of the log2 ratios obtained from microarray and Q-RT-PCR data at the same time points.

Exponentially growing BJ-TERT fibroblasts were transduced with control adenoviruses (Ad-CRE) and treated with 300 nM FVP for 0, 30', 1, 2, 4, 8, 16 and 24 h. The rational for transducing the cells with control adenoviruses in this experiment was that cells treated with FVP in the microarray experiments had previously been transduced with adenoviruses expressing no transgene to allow comparison to cells transduced with adenoviruses directing the expression of dnCDK9. After FVP treatment, BJ-TERT fibroblasts were collected at indicated time points and RNA was extracted. Q-RT-PCR analyses were then performed to determine transcript levels using Taqman probes specific for the genes listed above and represented as a fold change value of levels normalized to 1 at time zero (Figure 2A). The results from Q-RT-PCR and microarray analyses were comparable as it was shown that FOS, EGR1, JUNB and GADD45B were upregulated at late time points. However, they were all sharply and rapidly downregulated upon FVP treatment as early as 30 min (Figure 2A). CCND1 appeared to be upregulated without early downregulation. In contrast, MCL1 and HEXIM1 were downregulated with slower kinetics and remained down through the time course. As expected, there was no change in the expression of ALDOA.

To make the visualization of the comparison of microarray and Q-RT-PCR data clearer, Q-RT-PCR values from this experiment were converted to log2 ratios and represented with Java-TreeView with the same color gradient used in the microarray data (Figure 2B). This representation allows concise visualization and clusters the genes according to similar kinetics of expression. To compare the exact same time points in the microarray and Q-RT-PCR data, the microarray data and log2 converted Q-RT-PCR data for 4, 8, and 24 h of FVP treatment were visualized with Java-TreeView. Figure 2C demonstrates the similarity of the expression data obtained by microarray and Q-RT-PCR analysis. Next, this experiment was repeated under the same conditions, except that transduction with control adenoviruses was omitted. We found that the kinetics of downregulation/upregulation of all these genes was very similar (Additional file 2: Figure S2), indicating that viral transduction does not significantly affect the expression of these genes in BJ-TERT fibroblasts. Altogether these experiments demonstrate that several classical PRG/IRGs are rapidly downregulated but subsequently recover and are upregulated in the presence of FVP. Of note, these genes are upregulated at the same time that other genes are maximally downregulated (Figure 2B) and phosphorylation of the CTD of RNAPII on Ser-2 and Ser-5 remains inhibited (see below).

### Certain PRG/IRGs are significantly expressed in the presence of FVP during a mitogenic response

**Figure 3:**
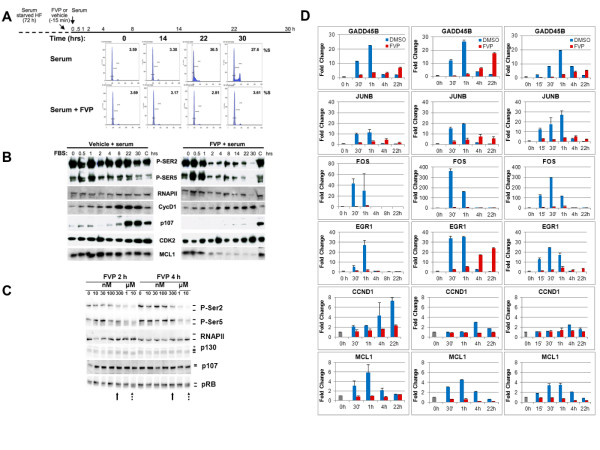
**Certain PRG/IRGs are significantly expressed in the presence of FVP during a mitogenic response. (A) **Effects of FVP in the cell cycle were determined by FACS of Propidium Iodide(PI)stained cells. The percent of cells in S phase is indicated at the top of each panel. BJ-TERT fibroblasts were treated with either DMSO or 300 nM FVP followed by serum stimulation for 0, 14, 22 and 30 h and stained with PI. **(B) **Western blot analysis of cell cycle progression, transcription markers and MCL1. BJ-TERT fibroblasts were harvested and lysed. Ten microgram of protein was resolved by 8% polyacrylamide/SDS gel electrophoresis, transferred to a PVDF membrane and specific antibodies were used to detect proteins, as indicated above. **(C) **300 nM FVP inhibits phosphorylation of the CTD of RNAPII on Ser-2 and Ser-5 without affecting the phosphorylation state of pocket proteins. Exponentially growing BJ-TERT fibroblasts were treated with FVP at 10 nM, 30 nM, 100 nM, 300 nM, 1 μM and 10 μM for 2 and 4 h. Whole cell lysates were resolved by 6% SDS/PAGE and immunoblotted with antibodies to the indicated proteins/phosphorylation sites. Solid and dashed arrows at the bottom indicate the concentration of FVP leading to dephosphorylation of RNAPII and pocket proteins, respectively. The asterisk indicates a crossreacting band recognized by the anti-p130 antibody. **(D) **mRNA levels of selected genes at the indicated time points were detected by Q-RT-PCR. The results of three different experiments are shown. Data are represented as a fold change value of levels normalized to 1 at time zero. The results of three different experiments are shown.

FOS, EGR1, JUNB and GADD45B are all known to be primary response genes to mitogenic stimuli as their transcripts accumulate in response to mitogenic stimuli in the absence of protein synthesis [21]. The experiments described in the previous section were done using exponentially growing BJ-TERT fibroblasts in the presence of complete medium with serum. To determine the effect of FVP in the expression of the PRG/IRGs in response to mitogenic stimulation, we performed experiments of serum starvation followed by mitogenic stimulation in the presence and absence of FVP. BJ-TERT fibroblasts were serum starved for three days with serum free medium. Serum starved BJ-TERT fibroblasts were treated either with 300 nM FVP or vehicle (DMSO) 15 min prior to serum stimulation. Following serum stimulation, cells were collected at the time points indicated in Figure 3.

First, we determined the effect of FVP upon serum stimulation of BJ-TERT cells on the cell cycle by flow cytometric analysis of propidium iodide stained cells and by western blot analysis of cell cycle markers (Figure 3A and 3B). Serum re-stimulated BJ-TERT cells exhibited 36% of cells in S phase 22 h post stimulation and cells reached the G2/M phases by 30 h. In contrast, cells re-stimulated with serum in the presence of FVP remained with a G0/G1 DNA content by 30 h. Thus, FVP completely blocked entry into S phase. For parallel western blot analysis, BJ-TERT fibroblasts were collected at additional time points as indicated. FVP inhibited expression of Cyclin D1, and completely blocked upregulation of p107, an E2F-dependent gene product. This indicates that cells are likely blocked prior to the restriction point in G1 or earlier, as passage through the restriction point is associated with activation of E2F dependent gene expression (Figure 3B) [23]. The effects of FVP in transcription were monitored by determining the phosphorylation state of RNAPII. CDK9 is thought to phosphorylate Ser-2, while Ser-5 is thought to be phosphorylated by CDK7 and other kinases. FVP potently inhibited Ser-2 and Ser-5 phosphorylation of RNAPII within 2 h of treatment, and importantly, phosphorylation remained inhibited through the time course, which is consistent with a general block in transcription. Also, consistently with the effects of FVP on the expression of MCL1 mRNA, we observed a block on MCL1 protein upregulation and sharp downregulation below its basal levels in quiescent cells (Figure 3B). To exclude the possibility that the concentration of FVP (300 nM) used in these experiments inhibited G1 CDKs, we determined the effects of FVP at different concentrations ranging from 10 nM to 10 μM on the phosphorylation of pocket proteins (pRB, p107 and p130), as compared to RNAPII CTD phosphorylation on Ser-2 and Ser-5 (Figure 3). In agreement with our previous work, 300 nM FVP resulted in inhibition of both Ser-2 and Ser-5 phosphorylation [15], while dephosphorylation of pocket proteins was only noticeable at a FVP concentration of 10 μM.

Next, we performed three independent serum starvation experiments to determine the effects of FVP on the transcript levels of various PRGs. BJ-TERT fibroblasts were serum starved for three days and subsequently stimulated with serum in the presence of FVP or vehicle (DMSO). Figure 3D shows rapid and potent serum-dependent stimulation of FOS, EGR1, JUNB and GADD45B, as would be expected from any highly inducible PRG in one of these three experiments. FOS and EGR1 peak at 30 to 60 min post stimulation, while GADD45B and JUNB peak at least 30 min later. Induction of these genes is more potent for FOS that reaches up to 350 fold, being near 10-30 fold for the other PRG genes. The expression of these four genes goes back to the expression level seen in quiescent cells between 4 and 22 h of serum stimulation. MCL1 behaves similarly, peaking about 1 h, but it is only upregulated 4 to 6 fold, while CCND1 peaks later and is upregulated to a lower extent (Figure 3D).

FVP inhibits the expression of all these genes. In the case of FOS, this inhibition is extremely potent, if not complete. MCL1 and CCND1 are also clearly inhibited, and in the case of MCL1, expression goes clearly below the basal levels observed in quiescent cells. Importantly, GADD45B, JUNB and EGR1 are all induced at significant levels in the presence of FVP, although there is clear variability within the experiments (Figure 3D). GADD45B is upregulated between 5 and 17 fold by 22 h, and induction over basal levels is first seen 1 h post stimulation. JUNB is induced 4-6 fold, peaking 4 h in the presence of FVP. On the other hand, EGR1 is the most variable; its expression is completely blocked by FVP in experiment 1 (Figure 3D, left panel), but potently upregulated in experiment 2 (Figure 3D, middle panel). These results suggest that the activities inhibited by FVP, which include CDK9, are clearly important for the mitogen induced transcription program. However, these results also show that the sensitivity of genes to FVP is highly variable and that some genes can be significantly expressed in the presence of FVP, when RNAPII phosphorylation is clearly inhibited.

### Some PRGs are transcribed in the presence of FVP

**Figure 4:**
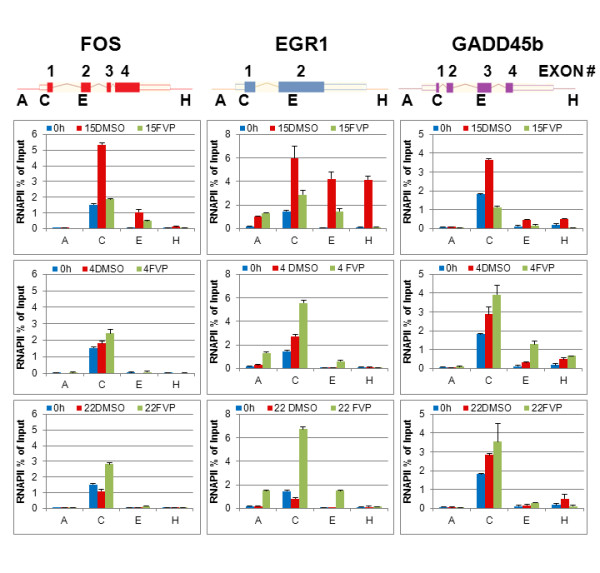
**Some PRGs are transcribed in the presence of FVP**. ChIP assays were performed, as described in the Methods section. Percent of input chromatin specifically immunoprecipitated with anti-RNAPII antibodies is represented at 0 and 15' (upper graph), 0 and 4 h (middle graph) and 0 and 22 h (bottom graph) for each primer set. The upper panel depicts the gene structure of FOS, EGR1 and GADD45B and the position of the corresponding primer pair sets (see text for details).

The experiments described above measure the expression of particular mRNAs, but they are not a direct measurement of transcription. The levels of any particular mRNA are the result of an equilibrium between RNA synthesis and degradation. Thus, it is conceivable that the induction in the expression of some PRG/IRGs is the result of potent RNA stabilization rather than effective transcription in the presence of FVP. To determine the direct effects of FVP in the presence of RNAPII in the transcribed region of FOS, EGR1 and GADD45 *in vivo*, we performed chromatin immunoprecipitation (ChIP) assays with anti-RNAPII antibodies. Localization of RNAPII near the promoter and in the body of the gene is a measurement of how actively a gene is being transcriptionally elongated by RNAPII [24-28]. BJ-TERT fibroblasts were serum starved for three days, followed by vehicle (DMSO) or 300 nM FVP treatment 30 min prior to serum stimulation. Following serum stimulation, cells were fixed in 1% formaldehyde/PBS and collected at 0, 15 min, 4 and 22 h. ChIPs were performed with antibodies to RNAPII and control IgGs, as described in the Methods section. The Q-PCR data is represented as a percentage of input chromatin amplified with each primer set (PS) in each ChIP. Four sets of primers were used for each selected PRG: a set of primers upstream of the promoter (A), a set downstream of the promoter just at the beginning of the first exon (C), a set in the body of the gene and a set presumably downstream of the gene (H) (Figure 4, top). Figure 4 shows the ChIP results for total RNA polymerase (RNAPII) at the FOS, EGR1 and GADD45B loci in the presence or absence of FVP. In the case of FOS, transcription elongation is not detected in serum starved quiescent cells, as no RNAPII is localized in the body of the gene (Figure 4, PS-E, absence of blue bar). A peak of RNAPII is localized downstream of the promoter (Figure 4, PS-C, blue bar) indicating that transcription is initiated but there is no elongation. Stimulation with serum for 15 min results in increased recruitment of RNAPII downstream of the promoter (Figure 4, left panel, PS-C, red bars). In addition, RNAPII is detected throughout the gene body (Figure 4, PS-E, red bars) 15 min post serum stimulation, indicating active transcription elongation. RNAPII remains bound near the promoter site at 4 and 22 h post serum stimulation, but RNAPII is not detected through the gene body, demonstrating that FOS is not transcriptionally elongated by RNAPII at 4 and 22 h post serum stimulation (Figure 4, left panel, PS-C and E). In the EGR1 gene, elongating RNAPII is also not detected in serum starved fibroblasts (Figure 4, middle panel, PS-E, absence of blue bar). However, EGR1 appears to be elongated by RNAPII 15 min post serum stimulation, as high levels of RNAPII are recruited to the body of this gene (Figure 4, middle panel, PS-E and H, red bars). Similarly to FOS, EGR1 is not elongated by RNAPII at 4 and 22 h post serum stimulation, as RNAPII is not detected in the gene body (Figure 4, middle panel, red bars, PS-E and H). In the case of GADD45B, there is little transcription detected in serum starved cells, but elongation by RNAPII appears to be detected 15 min post mitogenic stimulation (Figure 4, right panel, red bars, PS-E and H). In contrast to FOS and EGR1, GADD45B also appears to be elongated at 4 h of mitogenic stimulation, as RNAPII is detected with PS-E and H (Figure 4, right panel). Altogether confirms that these genes are highly inducible by mitogenic signaling, and subsequently downregulated to basal or near basal expression levels.

FVP inhibited elongation by RNAPII of FOS 15 min post serum stimulation by 2 fold, but elongation was completely blocked at 4 and 22 h (Figure 4 left panel, green bars, PS-E). Of note, RNAPII remained bound just downstream of the promoter at all times of FVP treatment, indicating that FVP does not prevent basal transcription initiation, although it eliminates the increased recruitment observed with serum (Figure 4, PS-C, compare three bars). In contrast to FOS, RNAPII is detected at all time points with PS-E of EGR1 upon FVP treatment, suggesting that EGR1 is transcriptionally elongated even in the presence of FVP. In addition, RNAPII recruitment is increased on EGR1 downstream of the promoter site at 4 and 22 h of FVP treatment (Figure 4, middle panel, green bars). GADD45B also appears to be transcriptionally elongated at 15 min, and 4 and 22 h of FVP treatment (Figure 4, right panel, green bars) but RNAPII levels are relatively much higher at 4 and 22 h of FVP treatment immediately downstream of the promoter.

In summary, FOS is transcriptionally elongated at early time points of serum stimulation but this gene is not elongated at later time points either in the presence or absence of FVP. In contrast, EGR1 and GADD45B are transcriptionally elongated at early time points upon serum stimulation; however, these genes are also transcriptionally elongated upon FVP treatment at all time points. Of note, these results are consistent with the mRNA expression effects of FVP in these genes as determined by microarray and Q-RT-PCR analysis. Considering that the expression of other genes is completely repressed and that cells treated with FVP exhibit potent inhibition of RNAPII, Ser-2 and Ser-5 phosphorylation (Figure 3B), it appears possible that both EGR1 and GADD45B posses alternative mechanisms of transcriptional elongation independent of CDK9 and other FVP-sensitive kinases.

## Discussion

Hierarchical clustering analysis of microarray expression ratios of select PRG/IRGs revealed that a subset of these genes were upregulated between 4 and 8 h of FVP treatment and maximally upregulated by 24 h. This at first appeared to be at odds with recently published work by Hargreaves et al (2009). The authors of this study have shown that stimulation of primary macrophages with LPS leads to rapid upregulation of a number of PRGs. They have shown that unstimulated PRGs exhibit paused RNAPII with Ser-5 phosphorylation at the promoters, but Secondary Response Genes (SRGs) do not. Their data also show that LPS stimulation leads to recruitment of CDK9 and Cyclin T1, and RNAPII with Ser-2 phosphorylation and this is blocked by DRB, a pharmacological inhibitor of CDK9. Our microarray analysis of PRG/IRGs has led to the identification of a cluster of about a third of these genes that are upregulated upon FVP treatment. This includes two classical PRGs, EGR1 and FOS that we selected for further study. Interestingly, genes in cluster 3 and 4 (Figure 1A) were found to be downregulated by 4 h of FVP treatment and subsequently upregulated, suggesting the possibility that PRGs are transiently downregulated by FVP.

Our q-RT-PCR data confirmed both the upregulation of genes in cluster 2 at late FVP time points and the hypothesis of the transient effects of FVP. In Figure 2A and 2B, it is clearly shown that EGR1, FOS, GADD45B and JUNB are rapidly downregulated by 30 min of treatment and go back to basal levels between 4 and 8 h, and then, are rapidly upregulated. One possible interpretation of these results is that FVP is at least partially metabolized in the cell, and thus, these genes are no longer inhibited. However, a number of observations argue against this possibility. First, RNAPII phosphorylation on Ser-2 and Ser-5 remains inhibited through the time course. Second, several PRG/IRGs remain inhibited through the time course. Third, genes in cluster 2 are not just going back to basal levels, but are clearly upregulated, often by several fold, under conditions in which housekeeping genes are not modulated. Fourth, previous work has shown genes that are transcribed independently of CDK9, such as certain p53 target genes [29] and the U2 snRNA and histone H2b genes [30].

To investigate this further, it was necessary to use a more defined model of primary response to stimulus. Since EGR1, FOS, JUNB and GADD45B are all responsive to serum stimulation [21,28,31], we selected this system. Our data clearly shows that FVP, at concentrations that do not inhibit pocket protein phosphorylation, completely blocks DNA synthesis and the upregulation of cell cycle markers, such as the expression of the E2F-dependent product p107, suggesting that cells are arrested before the restriction point in G1 or even earlier at the G0/G1 transition [23]. This arrest is preceded by potent inhibition of total Ser-2 and Ser-5 phosphorylation on the CTD of RNAPII, which remains low throughout the time course. Under these conditions, three genes (GADD45B, JUNB and EGR1) exhibited time dependent upregulation of their expression over untreated cells in the presence of FVP. Although there was variability between experiments, the trend was similar. In contrast, FOS expression was completely blocked under these conditions. Consistent with these results, RNAPII ChIP localization assays demonstrated the presence of RNAPII in the body of the EGR1 and GADD45B genes, at 4 and 24 h of serum stimulation in the presence of FVP. In contrast, RNAPII could not be detected in the body of the FOS gene at these time points. These data strongly suggest that the upregulation of EGR1 and GADD45B expression seen in the presence of FVP is due to active transcription. Another interesting observation is the accumulation of RNAPII in chromatin downstream of the promoter near the beginning of exon 1 of EGR1 and GADD45B. This accumulation can be due to increased recruitment of RNAPII under conditions of stress (sustained inhibition of transcription), but it could also indicate increased elongation of transcripts that is not fully productive. Distinguishing between these possibilities would require further experimentation addressing the recruitment of transcription factors and the phosphorylation of RNAPII using ChIP.

Two studies published when this work was being completed have examined the effects of pharmacological inhibition of CDK9 in the expression of PRGs. In the first of these studies, FVP dramatically inhibited the expression of FOS, EGR1, EGR2 and EGR3 upon 30 min of serum stimulation of HCT116 cells [28]. ChIP showed inhibition of RNAPII localization and diminished Ser-2 and Ser-5 phosphorylation of RNAPII in the body of the EGR1 and EGR2 genes. While this study did not determine the effects of FVP at later time points, they observed increased RNAPII at the promoter in the presence of FVP at 30 min post serum stimulation. This effect might be related to our observation of increased RNAPII near exon 1 of EGR1 gene at 4 and 24 h of serum stimulation. The second study utilized halogenated imidazole derivatives that blocked transcriptional elongation and, at least one of them, CDK9 activity [31]. In this study, both compounds potently inhibited the transcription of PRGs. However, the study did not analyze the effects of these compounds at later time points.

Two previous studies have investigated the rapid effects of FVP on gene expression globally. Both studies used concentrations of FVP of 1 μM. This concentration of FVP is very toxic, and it is unclear if any other CTD CDKs are inhibited under these conditions. Lam *et al. *showed that 1 μM FVP results in the rapid downregulation of genes up to 8 h [14]. Rahl *et al. *using ChIP-seq showed that 1 μM FVP completely blocks localization of RNAPII in the body of actively transcribed genes, but does not affect localization of RNAPII near the promoter [27]. If a concentration of 1 μM FVP is selective for CDK9 inhibition in cells, these data would mean that CDK9 is essential for transcriptional elongation of a large proportion of genes. Of note, Chao and Price showed previously that FVP treatment (300 nM) of HeLa cells inhibits transcription in *in vitro *run on assays by 70% [12]. Again, if 300 nM FVP selectively inhibits CDK9, these data would demonstrate that CDK9 inhibition halts transcription of most genes. This could be a direct effect on genes that require CDK9 for elongation, i.e. PRGs, and indirect on those genes not transcribed because of rapid depletion of inducible transcription factors.

Ser-2 and Ser-5 phosphorylation of the CTD of RNAPII have been linked to recruitment of histone chaperones such as Spt6 and/or histone modifications, including H3K36me3, which may play roles in nucleosome disassembly and assembly during elongation by RNAPII [32,33]. Thus, inhibition of RNAPII Ser-2 and Ser-5 phosphorylation at certain genes may prevent nucleosome reassembly and facilitate sustained transcription with a delay.

Finally, it is also conceivable that after a potent inhibition of transcriptional elongation that quickly depletes the cell from PRG/IRGs and other highly inducible transcription factors, the cell might require a boost in the expression of some of these PRG/IRGs. Gene expression might differentially oscillate before reaching a new transcriptional equilibrium. Some gene expression oscillation might be CDK9 independent, occurring while CDK9 is still effectively inhibited. Other changes might follow the expression of required PRG transcription factors. Understanding this fully will require more extensive studies using ChIP and/or ChIP-seq for RNAPII phosphorylated on Ser-2 and Ser-5, as well as recruitment of CDK9 and other RNAPII CDKs using FVP and more selective inhibitors of CDK9. Future gene expression profiling experiments should include short treatments with pharmacological inhibitors of CDK9. This will allow more precise identification of the groups of genes that are co-modulated in a time dependent manner.

Understanding the effects of CDK9 inhibition on gene expression is of considerable interest because of therapeutic implications. CDK9/Cyclin T1 activity is essential for HIV transcription, and hence, replication [1,13,34-37]. CDK9 activity is upregulated in hypertrophic cardiomyopathy [38]. Moreover, Mixed Lineage Leukemia [39] and certain ER-positive breast cancer [40,41] exhibit deregulation of P-TEFb activity. Thus, understanding the consequences of inhibiting CDK9 activity on gene expression over time is important. Our data suggest the possibility of a stress response to the effects of inhibiting transcriptional elongation that may result in the transcription of certain genes in a CDK9 independent manner. Some of the genes that would fit this category would be the PRG/IRG EGR1 and GADD45B, but the members of PRG/IRGs behaving in this manner are like to be larger.

## Conclusions

We have shown that certain PRGs are transcribed in the presence of FVP in a manner that appears independent of CDK9 suggesting the existence of a possible alternative mechanism for their transcription when P-TEFb kinase activity is pharmacologically inhibited. This could be part of a stress response to the effects of inhibiting transcriptional elongation. Understanding the effects of CDK9 inhibition on gene expression over time is of considerable interest because it is a potential therapeutic target in a number of diseases including AIDS, certain cancers, inflammatory processes and hypertrophic cardiomyopathy.

## Methods

### Cell culture and treatments

Human BJ-TERT fibroblasts are normal BJ fibroblasts immortalized with human Telomerase Reserve Transcriptase (hTERT) [42]. BJ-TERT fibroblasts were maintained in Dulbecco's Modification of Eagle's Medium (DMEM) (Cellgro) supplemented with 10% Fetal Bovine Serum (FBS) (Gemini) and 100 U/ml penicillin and 100 μg/ml streptomycin (Gemini). Cells were grown at 37°C in 5% CO_2_. For primary response gene assays, BJ-TERT fibroblasts were grown up to 50-70% confluence and starved in serum-free medium for 72 h as described in [43]. CDK9 was pharmacologically inhibited by adding Flavopiridol (FVP). FVP was prepared in DMSO at a concentration of 10 mM then dissolved in PBS to 100 μM and added to the medium to give a final concentration of 300 nM. Control cells were treated with the same concentration of DMSO (Dimethyl sulfoxide) dissolved in PBS. Thirty minutes after the addition of FVP or DMSO, serum starved cells were stimulated with serum and collected at the times indicated in the results section. Flow cytometric cell cycle analysis was done as previously described [43]. Where indicated, FVP treated cells were preinfected with control adenoviruses Ad-T-dnCDK9 plus Adeno-X™ Tet-Off™ in the presence of tetracyclin (dn + tet) to eliminate viral transduction as a variable in the comparison to dnCDK9 expressing cells (transduced with Ad-T-dnCDK9 plus Adeno-X™ Tet-Off™ in the absence of tetracyclin (dn)). BJ-TERT fibroblasts were infected with Ad-T-dnCDK9 plus Adeno-X™ Tet-Off™ adenviruses (dn) in the presence or absence of tetracyclin (tet) essentialy as previously descrived [15] and then treated with FVP for the times indicated in the figures.

### Protein analyses

Cells were lysed with ice-cold lysis buffer (50 mM Tris-HCl (pH 7.4), 5 mM EDTA, 250 mM NaCl, 50 mM NaF, 0.1% Triton X-100, 0.1 mM Na_3_VO_4_, 2 mM PMSF, 10 μg/ml leupeptin, 4 μg/ml aprotinin, and 40 μg/ml pepstanin). Proteins were resolved by 8% polyacrylamide/SDS gel electrophoresis, and transferred to a polyvinylidene difluoride (PVDF) membrane (Immobilon-FL, Millipore) in 10 mM CAPS/10% methanol buffer (pH 11). Western blot analyses were performed as described previously [15]. Bands were visualized by using Western Lighting Plus ECL reagent (Perkin Elmer) and X-Ray film or imaging with FluorChemQ Imaging System (Alpha Innotech). For Western blot analyses the following antibodies were used: Anti-RNAPII (A300-653A), anti-Ser-2 (A300-654A), and anti-Ser-5 (A300-655A) rabbit polyclonal antibodies were obtained from Bethyl. Anti-MCL1 (sc-819), anti-Cyclin D1 (sc-8396), anti-CDK2 (sc-163), anti-Cyclin E (sc-481), and anti-p107 (sc-318) rabbit polyclonal antibodies were obtained from Santa Cruz Biotechnology.

### Microarray cluster analysis

The Affymetrix Human Gene 1.0 ST transcript data set utilized in the cluster analysis described in this report will be reported elsewhere. Hierarchical cluster analysis (Cluster version 3.0, [44]) was performed to cluster genes using average linkage clustering and visualized with Java TreeView, version 1.0.13 as in [15].

### Q-RT-PCR and Q-PCR

RNA was isolated from BJ-TERT fibroblasts by using the RNeasy Extraction Kit (Qiagen). RNA expression was determined in 96 well plates using the TaqMan 1 Step PCR Kit (Applied Biosystems) and 20 μl reactions containing specific TaqMan probes. mRNA levels were determined using an ABI Prism 7000 RT-PCR system (Applied Biosystems). Taqman probes used in this study are as follow: JUNB (Hs00357891_s1), ALDOA (Hs00605108_g1), HEXIM1 (Hs0053918_s1), GADD45B (Hs00169387_m1), CCND1 (Hs99999004_m1), MCL1 (Hs03043899_m1), FOS (Hs01119256_g1), EGR1 (Hs00152928_m1). A GAPDH probe was used for normalization. Threshold Cycle (CT) values for each sample were normalized with GAPDH CT values. Then, a second normalization was performed by subtracting normalized values of each time point from the control normalized value per each gene.

SYBR Green qPCR Mix (Fermentas) was used to determine relative DNA amounts in input chromatin samples and ChIPs (anti-RNAPII) and IP mock (IgG) in 96 well plates using the StepOnePlus PCR instrument (Applied Biosystems). RNAPII IP and mock ChIP CT values were normalized with input CT values to obtain the percentage of input values separately for each primer set. The percentage of input was calculated, mock ChIP values were subtracted from RNAPII ChIP values and results were visualized via Excel graphs.

Additional file 3: Table S1 shows primers for FOS and EGR1 (Donner, et al, 2010), and GADD45B genes. The Reference Sequence (RefSeq) of human GADD45B was obtained from ECR browser http://ecrbrowser.dcode.org. Four different primer pairs were designed using Oligo Perfect™ Designer http://www.invitrogen.com to amplify the following sequences: Primer set A: upstream of the gene; Primer set C: 5'UTR close to Exon 1; Primer set E: Exon 3; Primer set H: downstream of the gene.

### Chromatin immunoprecipitation (ChIP)

ChIP experiments were carried out as described in [28]. BJ-TERT fibroblasts were grown in 10 cm plates up to 50-70% confluence. Serum-starved cells were treated with FVP or DMSO, and then stimulated with serum as described in the cell culture section. Cells were fixed with 1% formaldehyde/PBS for 15 min at room temperature. The cross-linking reaction was stopped by adding glycine (0.125 M). Cells were then washed twice with cold PBS, and harvested with complete Szak's RIPA buffer (150 mM NaCl, 1% Nonidet P-40, 0.5% deoxycholate, 0.1% SDS, 50 mM Tris-HCl pH 8, 5 mM EDTA, Protease Inhibitor Cocktail (Roche), 10 mM PMSF). Cells were then sonicated for 30 cycles of 15 s, followed by 1 min rest on ice (50% duty cycle). Sonicated chromatin was cleared by centrifugation. The amount of protein in the cell was quantified by using the BCA Protein assay kit (Thermo Scientific). Sonicated DNA was about 300 bp. Sheared chromatin extracts were pre-cleared with Protein A sepharose beads. Protein A beads loaded with antibodies and blocked with BSA and Salmon Sperm DNA were incubated with chromatin overnight at 4°C on a rotator. The immunoprecipitated chromatin was washed twice with incomplete RIPA buffer (without PMSF and Protease Inhibitor cocktail), four times with Szak IP Wash Buffer (100 mM Tris HCl pH 8.5, 500 mM LiCl, 1% Nonidet P-40, 1% deoxycholate), then twice again with incomplete RIPA buffer and twice with cold 1X TE. The cross-links were then reversed by adding Talianidis Elution Buffer (1.5X) (70 mM Tris HCl pH 8, 1 mM EDTA, 1.5% SDS), and NaCl (200 mM final) overnight at 65°C. Samples were then treated with 20 μg of Proteinase K (Qiagen) for 30 min at 45°C, extracted with Phenol/Chloroform and ethanol precipitated. The pellet was resuspended in water and analyzed by Q-PCR as described above. The following antibodies were used for ChIP: Anti-RNAPII (8WG16) monoclonal antibodies from Covance and mouse monoclonal IgGs (sc-2025) from Santa Cruz Biotechnology.

## Competing interests

The authors declare that they have no competing interests.

## Authors' contributions

HK performed the cellular treatments and analyses shown in Figures 2, 3 and 4 and prepared a first draft of those figures and the results section of the manuscript. JG and XG performed the microarray analysis used for the analysis of the expression of PRGs in cells treated with FVP or expressing dnCDK9. JG oversaw the cell culture experimental strategies. DG oversaw and discussed ChIP experiments. XG designed the experiments, analyzed the data and wrote the manuscript. All the authors discussed the results and read, revised and approved the final manuscript.

## Supplementary Material

Additional file 1 **Figure S1**. The highest correlation between downregulated PRGs by dnCDK9 and FVP occurs in the cluster 1 of PRGII. Hierarchical clustering of both genes and gene arrays was performed for the indicated PRG-I (left) and PRG-II (right) clusters designated in Figure 1A, and visualized with Java TreeView. The array node correlation is shown adjacent to each node (blue digits). A color gradient legend indicates fold changes in expression. Click here for file

Additional file 2 **Figure S2**. The Biphasic effect of FVP in the expression of certain PRG/IRGs in BJ-TERT fibroblasts is independent of whether cells are preinfected with control adenoviruses. (A) BJ-TERT fibroblasts were treated with 300 nM FVP and the expression of indicated mRNAs was determined by Q-RT-PCR analysis. Data was represented as a fold change value with respect to mRNA expression at time zero essentially as described in Figure 2A. Click here for file

Additional file 3 **Table S1**. Forward and Reverse Primers for ChIP Analyses for FOS and EGR1 genes (Donner et al, 2010) and the GADD45b Gene. Click here for file
