# Supplementary figures and images for: Complex effects of flavopiridol on the expression of primary response genes
Source: Cell Div. 2012 Mar 29;7:11. doi: 10.1186/1747-1028-7-11 (PMC3339560; doi:10.1186/1747-1028-7-11)

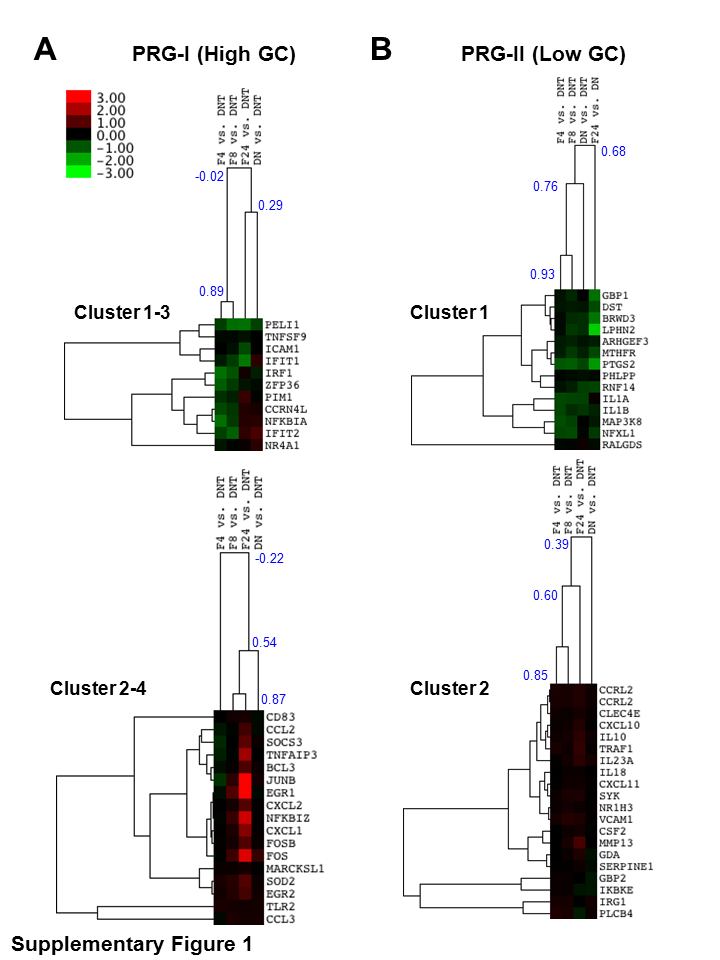

Supplement: Additional file 1 — Figure S1. The highest correlation between downregulated PRGs by dnCDK9 and FVP occurs in the cluster 1 of PRGII. Hierarchical clustering of both genes and gene arrays was performed for the indicated PRG-I (left) and PRG-II (right) clusters designated in Figure 1A, and visualized with Java TreeView. The array node correlation is shown adjacent to each node (blue digits). A color gradient legend indicates fold changes in expression. [file 1747-1028-7-11-S1.TIFF]

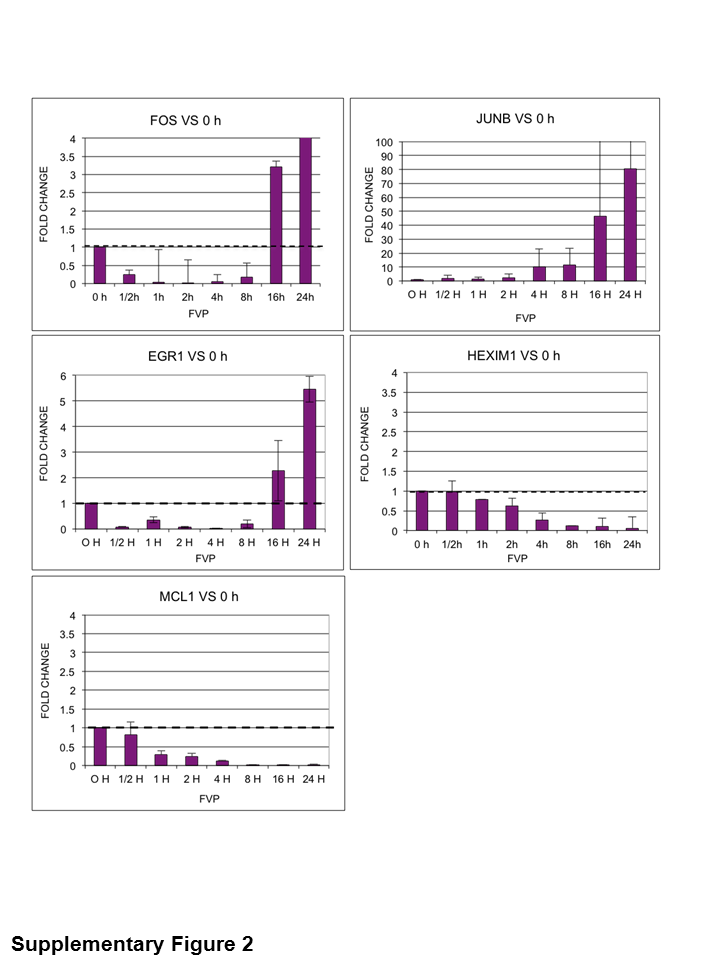

Supplement: Additional file 2 — Figure S2. The Biphasic effect of FVP in the expression of certain PRG/IRGs in BJ-TERT fibroblasts is independent of whether cells are preinfected with control adenoviruses. (A) BJ-TERT fibroblasts were treated with 300 nM FVP and the expression of indicated mRNAs was determined by Q-RT-PCR analysis. Data was represented as a fold change value with respect to mRNA expression at time zero essentially as described in Figure 2A. [file 1747-1028-7-11-S2.TIFF]
