# Supplementary material for: Complex effects of flavopiridol on the expression of primary response genes
Source: Cell Div. 2012 Mar 29;7:11. doi: 10.1186/1747-1028-7-11 (PMC3339560; doi:10.1186/1747-1028-7-11)
Supplement: Additional file 3 — Table S1. Forward and Reverse Primers for ChIP Analyses for FOS and EGR1 genes (Donner et al, 2010) and the GADD45b Gene. [file 1747-1028-7-11-S3.DOCX]

**Supplementary Table 1. Forward and Reverse Primers for ChIP Analyses for FOS and EGR1 genes (Donner et al, 2010) and the GADD45b Gene.**

| GENE | Amplicon  Name | Forward Primer | Reverse Primer |
| --- | --- | --- | --- |
| FOS | A | GATCTAGTTGTGAATGGCAGTCATG | TCAAGCTTTGAATTCCTGAGTCTG |
|  | C | TGAGCCCGTGACGTTTAC | TGCAGATGCGGTTGGAG |
|  | E | CGTCTCCAGTGCCAACTTCA | CCGGACTGGTCGAGATGG |
|  | H | TCCTTCCAAATCCCTTTTACTCAC | AAATTTCCCAGAGTTAATAATCCCTG |
| EGR1 | A | AGTGGCCGTGACTTCCTATCC | CTCGATCTATGGCACGGTGTC |
|  | C | ACCCGTGCCGTTCCAGA | ATCTCTCGCGACTCCCCG |
|  | E | CTGCCCCCCATCACCTATACT | CCACAAGGTGTTGCCACTGTT |
|  | H | CAGAGGAACAATGAGGTATCCCC | CACTTCACTCAGGGCCTGATAAC |
| GAD45B | A | GTCGCAGCCAGGAGAGACT | GTTCTGCTGGGGGTGGAG |
|  | C | GAAGGTTTTGGGCTCTCTGG | AGCTCTTCCAGCGTCATGTT |
|  | E | CAGACAGCGTGGTCCTCTG | TGTCACAGCAGAAGGACTGG |
|  | H | GGCACCTGGTACCACACAG | GCCTATCCTGGGATGGAGAA |
